# Anévrysme de l'aorte thoracique d'origine traumatique: cas clinique suspect

**DOI:** 10.11604/pamj.2015.21.241.6484

**Published:** 2015-08-04

**Authors:** Faustin Mbangi Bontolo, Pierre Mols, Pierre Youatou, Ahmed Sabri Ramadan, William Ngatchou

**Affiliations:** 1 CHU Saint Pierre, Bruxelles, Belgique

**Keywords:** Aorte, traumatisme thoracique, anévrysme, Aorta, chest trauma, aneurysm

## Abstract

Dans ce travail nous rapportons le cas d'un homme d'origine africaine du nord, âgé de 51 ans, qui s'est présenté à l'urgence pour des douleurs thoraciques constantes depuis un jour. Dans son anamnèse on note un enrouement de la voix depuis deux mois, une notion d'accident de circulation il y a environ dix ans. Patient sportif, fait de la boxe et travaille comme agent de sécurité dans une boite de nuit. La radiographie du thorax et l'angio-scanner thoracique montrent un volumineux anévrisme non compliqué de la crosse et du tiers distal de l'aorte thoracique descendante (7cmx7.8cm en vue axiale). Le patient a bénéficié d'une cure chirurgicale de ce volumineux anévrisme de l'aorte thoracique. Nous discutons des étiologies, des mesures cliniques et para cliniques qui permet le diagnostic de cette entité clinique rare pouvant être une erreur diagnostique pour un médecin urgentiste.

## Introduction

L'anévrisme de l'aorte thoracique (AAT) est une dilatation permanente et localisée de l'artère, de plus de 50% par rapport au diamètre normal, avec une perte de parallélisme de ses bords en forme de sac (Anévrysme sacciforme) ou de fuseau (Anévrysme fusiforme) [1]. Une dilatation de moins de 50% du diamètre normal est une ectasie [1].

L'anévrisme peut se rencontrer sur toutes les portions de l'aorte. La répartition anatomique est la suivante: aorte ascendante 40%, crosse de l'aorte 10%, aorte descendante 50% [1, 2]. L'AAT est une affection relativement rare, de fréquence variable de 2,5 à 3,4% selon les séries [3, 4]. Son incidence et sa prévalence sont en augmentation. On estime son incidence à 5,9/100000/habitants/an [3, 4]. Il existe plusieurs étiologies à un anévrisme aortique. Les lésions dégénératives de l'athérome sont les étiologies les plus fréquemment rencontrées, mais les AAT peuvent avoir d'autres origines telles que la média nécrose d'origine génétique (Marfan, Ehlers-Danlos), l'aortite, traumatisme et l'anévrisme mycotique [5, 6]. Il existe une étiologie dominante pour chaque topographie: atteinte dystrophique du tissu élastique pour l'aorte ascendante, post-traumatique pour l'aorte horizontale (crosse), athérome pour l'aorte descendante [5, 6].

La plupart des personnes qui ont un AAT sont asymptomatiques. Le diagnostic étant fait fortuitement lors d'une radiographie du thorax ou d'une échographie effectuée pour une autre pathologie cardiaque ou pratiquée de façon systématique dans un contexte familial tel que la maladie de Marfan [4–6]. L'examen physique est peu spécifique; néanmoins on peut observer un souffle diastolique en cas d'insuffisance aortique, ou des signes cliniques d'insuffisance cardiaque [4–6]. Le bilan préconisé est une radiographie du thorax, une échographie et l'angio-scanner thoracique [4–6]. L'indication opératoire est posée pour un diamètre de plus de 55 mm dans l'aorte ascendante et la crosse (plus de 50 mm en cas de syndrome de Marfan) et de plus de 60 mm dans l'aorte descendante [6]. Les autres indications au traitement sont l'apparition des symptômes (compression d'un organe de voisinage), anévrisme compliqué (fissuration, rupture) ou une croissance annuelle supérieure à 10 mm. Ces anévrismes conduisant à une chirurgie sans délai [6].

## Patient et observation

Un homme de 51 ans, d'origine nord africaine, sportif (boxeur) se présente au service des Urgences pour des douleurs thoraciques constantes depuis un jour avec notion d'enrouement de la voix depuis environ deux mois. Le patient avait été victime d'un grave accident de circulation il y a environ dix ans (choc frontal, alors qu'il était conducteur d'un véhicule). Il fume environ 20 cigarettes par jour et n'a aucun antécédent cardiovasculaire personnel ou familial connu. L'examen clinique montre un patient avec un bon état général, eupnéique au repos et normocoloré.

**Figure 1:**
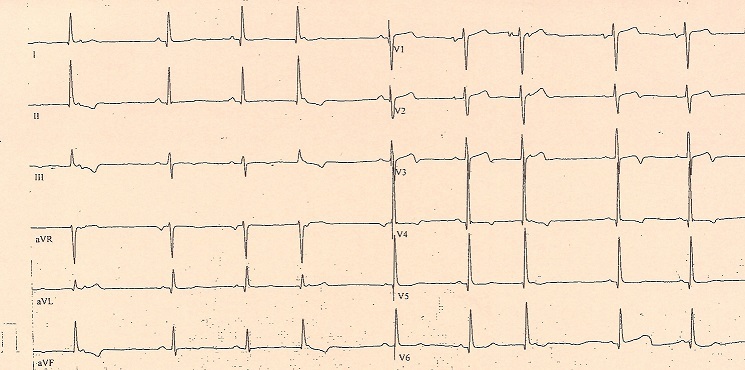
ECG: rythme sinusal avec extrasystoles auriculaires, fréquence à 55 par minute, altération de la repolarisation inférieure et antérolatérale compatible avec une maladie cardiaque

Sa tension artérielle brachiale est de 160/110 mmHg à droite et 145/100 mmHg à gauche. Il a une fréquence cardiaque de 70 battements par minute, une température mesurée à 36,8°C et une saturation en oxygène de 100% à l'air ambiant. L'auscultation cardio-pulmonaire est normale. L’électrocardiogramme (Figure 1) montre un rythme sinusal avec quelques extrasystoles auriculaires, une fréquence à 55 par minutes et des ondes T négatives en inférieur et en antérolatéral compatibles avec une maladie cardiaque.

**Figure 2:**
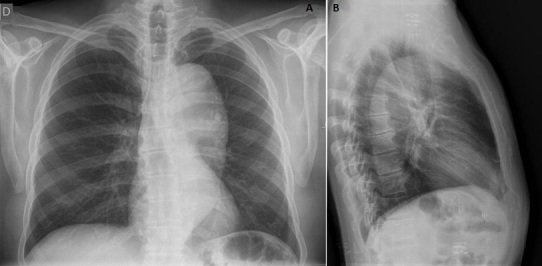
Rx thorax: élargissement de l'aorte thoracique estimé à 9 cm avec déroulement de l'aorte, vue de face, A) vue de face; B) vue de profil

**Figure 3:**
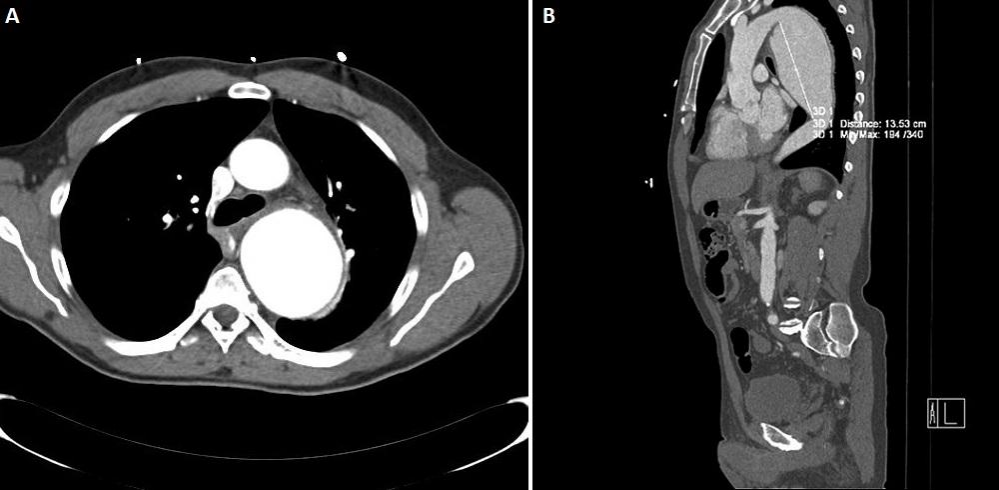
Angio-scanner thorax: élargissement de l'aorte thoracique estimé à 9 cm avec déroulement de l'aorte (A: coupe axiale: diamètre de 78x70 mm. En reconstruction para sagittale l'anévrisme mesure 14 mm de long. Le versant postérieur de la crosse de l'aorte pré-anévrismal mesure 30mm et 29 mm au niveau du tiers distal de l'aorte thoracique descendante; B: coupe sagittale: volumineux anévrisme du versant postérieur de la crosse de l'aorte et de la moitié supérieure de l'aorte thoracique descendante)

Le bilan sanguin montre un examen hématologique et un ionogramme normaux. La troponine T ultrasensible au temps zéro et après 3heures est normale. La radiographie du thorax de face (Figure 2) montre un élargissement de l'aorte thoracique estimé à 90 mm avec déroulement de l'aorte justifiant la réalisation d'un angio scanner total de l'aorte (Figure 3) montrant la présence d'un volumineux anévrisme du versant postérieur de la crosse de l'aorte et de la moitié supérieure de l'aorte thoracique descendante. Il n'y a pas de thrombose ou de dissection. En coupe axiale (Figure 3, A) il montre un diamètre de 78 x 70 mm. En reconstruction para-sagittale l'anévrisme mesure 14 cm de long. Le versant postérieur de la crosse de l'aorte pré-anévrismal mesure 30 mm et 29 mm au niveau du tiers distal de l'aorte thoracique descendante.

Le traitement aux urgences a consisté à l'administration de sufentanyl et de nicardipine en intraveineux. Le patient a été transféré aux soins intensifs et opéré en semi urgence deux jours plus tard. L'intervention a consisté en une résection de l'anévrisme sous circulation extracorporelle et à l'interposition d'une prothèse vasculaire en dacron. L’évolution postopératoire était satisfaisante. L'examen microscopique de la pièce opératoire était compatible avec l'athérosclérose modérée. L'intima était épaissie et présentait un thrombus récanalisé. L'hypothèse post-traumatique ancien est évoqué.

## Discussion

L'intérêt de notre cas clinique est de rappeler l'importance pour les médecins urgentistes d'inclure l'AAT et ses complications possibles dans le diagnostic différentiel des douleurs thoraciques.

Dans notre démarche diagnostique nous avons exclu par l'ECG et le dosage répété de Troponine le syndrome coronarien aigu qui aurait pu être un diagnostic chez ce patient. Les autres causes fréquentes de douleurs thoraciques (pneumothorax, foyer pulmonaire…) ayant été exclues par l'anamnèse, l'examen physique et la radiographie du thorax. L'enrouement de la voix et la douleur thoracique nous ont fait penser à un processus expansif au sein du médiastin, diagnostic finalement confirmé par la radiographie du thorax qui montrait un volumineux anévrisme de l'aorte thoracique.

Trois grandes étiologies dominent la pathologie anévrismale de l'aorte thoracique: l'athérosclérose (<72 à 92%) des cas selon Culliford et coll [7] et Moreno Cabral [8], les dystrophies du tissu élastique, et le traumatisme dans 11% des cas [7, 8]. N'ayant pas d'iconographie aortique du patient avant l'accident de roulage décrit comme violent 10 ans auparavant, l'origine traumatique n'est qu'une hypothèse. Mis à part le tabagisme modéré, le patient ne présentait aucun facteur de risque cardiovasculaire. La pièce opératoire montrait une athéromatose modérée avec une zone intimale présentant un repli contenant un thrombus faisant suspecter aux anatomopathologistes une lésion ancienne. Nolan et al [9] ont rapporté une série de 400 cas d'anévrisme traumatique de l'aorte thoracique dont 85% étaient devenus symptomatiques après 45 ans d’évolution. Alain Verdant [10] a présenté une série personnelle de 52 cas d'anévrisme thoracique traités en moyenne 14 ans après leur accident, 85% des malades souffraient d'un syndrome douloureux ou compressif (toux, dyspnée, dysphagie, raucité de la voix) comme dans notre cas clinique. Le morphotype et l'histoire familiale de notre patient ne faisaient pas penser à une origine génétique.

Bien que rare, la syphilis est une des causes décrites de l'anévrisme de l'aorte thoracique, la sérologie syphilitique n'a pas été demandée vu le contexte peu suspect. La plupart des patients avec l'AAT sont asymptomatiques. Notre patient a consulté pour des douleurs thoraciques et un enrouement de la voix. Une douleur d'origine anévrismale est toujours très significative d'un danger imminent pour une lésion habituellement asymptomatique. Elle traduit soit un remaniement soudain de la paroi aortique, soit une sur distension pariétale lors d'une crise hypertensive ou encore l'initiation d'une fissure sans fuite sanguine suffisante pour s'accompagner des changements hémodynamiques. Notre patient avait à l'admission une Tension artérielle de 160/110 mmHg à droite et 145/100 mmHg à gauche. Cette hypertension artérielle pourrait favoriser une sur distension de la paroi anévrismale à l'origine de douleur thoracique ressentie par le patient. L'enrouement de sa voix pourrait être dû à une compression du nerf récurent.

L'exploration des anévrismes a bénéficié ces dernières années de progrès de l'imagerie médicale. La radiographie du thorax et l’échographie trans-thoracique voire trans-oesophagienne sont des examens de première intention [6]. L'angio-scanner thoracique est indispensable en préopératoire, il permet de faire une cartographie des anévrismes de l'aorte thoracique, ainsi que le bilan d'extension artérielle [6, 11]. Il permet aussi une orientation du diagnostic étiologique et la recherche des complications (fissure, rupture). La place de l'IRM est devenue prépondérante devant son caractère peu irradiant et peu invasif permettant d’être répétée lors du suivi du patient [6].

Le traitement médical par bêta-bloquants et un suivi échocardiographique (1 à 2 fois par an) sont recommandés pour les patients asymptomatiques [2, 6]. Les bêta-bloquants ont démontré un effet freinateur sur la dilatation de la racine aortique et améliorent la survie surtout dans le cas de syndrome de Marfan [6]. L'utilisation des inhibiteurs de l'enzyme de conversion pourrait permettre de réduire le risque de rupture d'AAT [12]. Chez les malades asymptomatiques, le diamètre de l'anévrisme est le meilleur critère pour décider de l'intervention chirurgicale. Le risque de rupture est important pour les anévrismes de taille de 60 mm pour l'aorte ascendante et de 70 mm pour l'aorte descendante [4–6]. Une intervention est recommandée sur l'aorte ascendante lorsque le diamètre est supérieur ou égal 55 mm [6, 9]. A noter que certains facteurs favorisent des complications pour des diamètres moindres et justifient une intervention chirurgicale plus précoce: maladie de Marfan, histoire familiale de dissection aortique, valve aortique bicuspide [6]. Une intervention chirurgicale est recommandée sur l'aorte descendante lorsque le diamètre est supérieur ou égal à 65 mm, cette recommandation passant 60 en cas de maladie de Marfan [6]. Tous les anévrismes thoraciques symptomatiques ou compliqués doivent être opérés [6]. Cette pathologie est potentiellement mortelle surtout en cas de rupture, complication gravissime et souvent fatale dont la mortalité globale dépasse 90%. Il est donc important d'opérer avant la survenue d'une dissection ou d'une rupture [6].

## Conclusion

L'anévrisme de l'aorte thoracique est une pathologie rare habituellement asymptomatique et constitue une menace permanente d'erreur diagnostique pour le médecin urgentiste. Les anévrismes traumatiques de l'aorte peuvent devenir symptomatiques après plusieurs années d’évolution. La cure chirurgicale ne devra pas attendre les complications étant donné le taux de mortalité élevé dans ce contexte.
